# Supplementary material for: Diversity of Mycobacterium tuberculosis across Evolutionary Scales
Source: PLoS Pathog. 2015 Nov 12;11(11):e1005257. doi: 10.1371/journal.ppat.1005257 (PMC4642946; doi:10.1371/journal.ppat.1005257)
Supplement: S3 Table — Reference-guided assemblies for within-host samples were sub-sampled without replacement to a uniform coverage of 50X. Sliding-window analyses of π and ϴW were performed with default PoPoolation equations that account for sequencing error in pooled-data [27]. A window-size of 100Kb, a step-size of 10Kb, and a “pool-size” of 10,000 were used (see S1 File for justification). Estimates for within-host samples were generated under three parameter sets: mbq20 –a “minimum base quality score” of 20 with a “minimum minor allele count” of 2; mbq30 –a “minimum base quality score” of 30 with a “minimum minor allele count” of 2; mc3 –a “minimum base quality score” of 20 with a “minimum minor allele count” of 3. Genome-wide estimates are expressed as the mean across all windows covered by at least 60% under the given parameters. Genome-wide estimates for the global and lineage-specific datasets were performed with classical equations in PoPoolation (“disable corrections” flag enabled). Only sites covered by >75% of strains were included in the analyses. A window-size of 100Kb and a step-size of 10Kb were used, and genome-wide estimates are expressed as the mean across all windows passing criteria. Other parameters were not applicable to between-host datasets. (PDF) [file ppat.1005257.s003.pdf]

Population		π			θ		
Between-host	***n***		**GATK**			**GATK**	
Global	201		2.34E-04			1.01E-03	
Lineage 2	37		5.37E-05			1.59E-04	
Lineage 4	53		1.19E-04			3.39E-04	
Within-host		**mbq20**	**mbq30**	**mc3**	**mbq20**	**mbq30**	**mc3**
Patient A							
0 month		1.87E-05	NA	3.33E-06	5.92E-05	NA	5.24E-06
19 month		2.96E-05	5.88E-06	5.39E-06	9.65E-05	1.35E-05	1.18E-05
24 month		4.43E-05	1.82E-05	1.96E-05	1.23E-04	4.32E-05	3.91E-05
Patient B							
35 month		4.90E-04	4.96E-04	3.23E-05	1.69E-03	1.72E-03	7.91E-05
Patient C							
-1 month		6.94E-05	1.17E-05	2.11E-05	2.10E-04	2.63E-05	4.29E-05
11 month		4.99E-04	5.66E-04	2.21E-05	1.73E-03	1.97E-03	5.72E-05
Patient D							
0 month		1.75E-05	6.51E-06	8.53E-06	4.77E-05	1.66E-05	1.75E-05
9 month		2.06E-05	8.09E-06	1.15E-05	5.36E-05	2.05E-05	2.31E-05
11 month		3.39E-05	1.34E-05	1.85E-05	8.91E-05	3.53E-05	3.76E-05
20 month		1.48E-05	6.00E-06	6.67E-06	4.18E-05	1.64E-05	1.41E-05
Patient E							
0 month		2.53E-05	1.99E-05	2.09E-06	8.56E-05	6.69E-05	3.60E-06
8 month		2.40E-05	1.88E-05	1.87E-06	8.17E-05	6.35E-05	3.54E-06
12 month		2.63E-05	2.00E-05	1.96E-06	8.99E-05	6.82E-05	4.02E-06
14 month		2.48E-05	1.85E-05	2.28E-06	8.37E-05	6.25E-05	4.34E-06
28 month		2.63E-05	2.00E-05	2.85E-06	8.68E-05	6.49E-05	4.05E-06
31 month		2.71E-05	2.17E-05	2.91E-06	8.98E-05	7.09E-05	4.44E-06
34 month		2.43E-05	1.88E-05	2.67E-06	8.06E-05	6.14E-05	4.39E-06
39 month		2.35E-05	1.74E-05	2.47E-06	7.81E-05	5.72E-05	3.86E-06
42 month		2.39E-05	1.81E-05	2.27E-06	7.97E-05	6.02E-05	3.42E-06
