# Supplementary material for: Diversity of Mycobacterium tuberculosis across Evolutionary Scales
Source: PLoS Pathog. 2015 Nov 12;11(11):e1005257. doi: 10.1371/journal.ppat.1005257 (PMC4642946; doi:10.1371/journal.ppat.1005257)
Supplement: S1 File — Supplementary information on our sensitivity analysis of the PoPoolation Software [27]. This document includes justification of our parameter choices, as well as four figures with legends in the document. (PDF) [file ppat.1005257.s010.pdf]

S1 File for “Diversity of *Mycobacterium tuberculosis* across evolutionary scales”

Table of Contents

Note S1 Justification of parameter choices for PoPoolation software 2
Fig 1 Effect of “pool-size” on PoPoolation calculations 3
Fig 2 Effect of coverage on PoPoolation calculations 4
Fig 3 Effect of the minimum minor allele count on PoPoolation calculations 5
Fig 4 Effect of “window-size” on PoPoolation calculations 6

Note S1. Justification of parameter choices for PoPoolation software.

We sought to determine the effect of input parameters on the estimators implemented in the PoPoolation package to calculate nucleotide diversity (π), Watterson's theta (Θ_W), and Tajima's D (Kofler et al. 2011). Colony forming unit (CFUs) counts of *Mycobacterium tuberculosis* (*M.tb*) from the sputum samples collected were not available. Due to this uncertainty in the number of individuals in the population sample sequenced ("pool-size") we explored the effects of varying pool-size on calculations, and searched the literature for average CFU counts of *M.tb* from sputum samples. We tested the following values of pool-size: 60, 1000, 10000, 100000, and 1000000 (Fig 1). Pool-sizes above 10,000 yielded the same value of π and Θ_W as that calculated with a pool-size of 10,000, and the software was unable to calculate Tajima's D with pool-sizes set to 100,000 and 1,000,000. Average CFUs of *M.tb* from TB patients' initial sputum samples are consistently >10,000 across multiple studies (M.L. Joloba et al. 2000; Palaci et al. 2007; Bark et al. 2011). M.L. Joloba et al. (2000) found mean pretreatment sputum bacillary load of non-HIV-infected patients to be 5.58 ± 0.68 $\log_{10}$ CFU/ml. Similarly, Palaci et al. (2007) found that the mean bacillary loads of cavitary TB patients' sputum samples was 5.2 ± 1.4 $\log_{10}$ CFU/ml. Bark et al. (2011) report similar counts with the median bacillary load of sputum samples being 6.2 $\log_{10}$ CFU/ml (IQR 5.6 to 6.7). Bacillary loads have been shown to decrease over the course of treatment (M.L. Joloba et al. 2000). However, all samples examined in the current study are from individuals with extensive TB indicated by the acquisition of drug resistance, culture positivity, and treatment outcomes, and it has been shown that bacillary load increases on average with disease severity (Palaci et al. 2007). Given that measures of diversity did not change when the pool-size was increased beyond 10,000, and that the average estimates of CFUs from TB patients' sputum samples were on the order of 10^5-10^6, we used 10,000 as the pool-size for all of our analyses.

Coverage and the minimum minor allele count ("mc") were found to affect calculations (Fig 2, Fig 3). Due to the spread in average coverage between samples (ranging from 56X - 191X after filtering), we decided it best to sub-sample sequence data to a uniform coverage of 50X for each sample. The developers of PoPoolation suggest a minimum minor allele count of 2 for 50X coverage with their estimators, and have documented error rates based on artificial data for these parameters (Kofler et al. 2011); we report results using these parameters in the main text and include calculations performed with varying parameters in the supplement. Window-size did not affect the mean of estimates, only the variance within each sliding-window (Fig 4). We thus report all population estimators as the mean of each statistic calculated across 9 replicate sub-samplings of 50X.

References:

Bark CM et al. 2011. Time to detection of Mycobacterium tuberculosis as an alternative to quantitative cultures. Tuberculosis. 91:257–259. doi: 10.1016/j.tube.2011.01.004.

Kofler R et al. 2011. PoPoolation: a toolbox for population genetic analysis of next generation sequencing data from pooled individuals. PloS One. 6:e15925. doi: 10.1371/journal.pone.0015925.

M.L. Joloba et al. 2000. Quantitative sputum bacillary load during rifampin-containing short course chemotherapy in human immunodeficiency virus-infected and non-infected adults with pulmonary tuberculosis. Int. J. Tuberc. Lung Dis. 4:528–536.

Palaci M et al. 2007. Cavitary Disease and Quantitative Sputum Bacillary Load in Cases of Pulmonary Tuberculosis. J. Clin. Microbiol. 45:4064–4066. doi: 10.1128/JCM.01780-07.

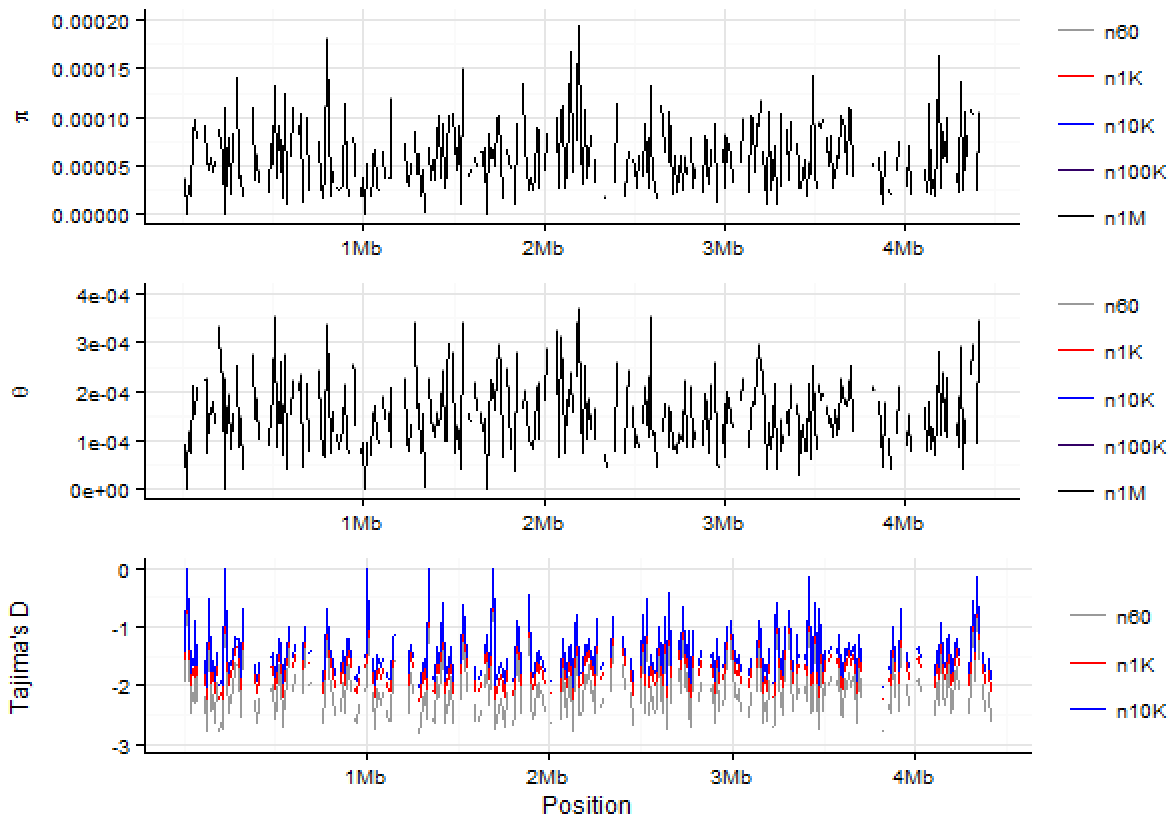

Fig 1. Effect of "pool-size" on PoPoolation calculations. Calculations were performed using a window-size of 10,000, and a minimum minor allele count of 2. Calculations were performed varying pool-size as indicated in the figure key.

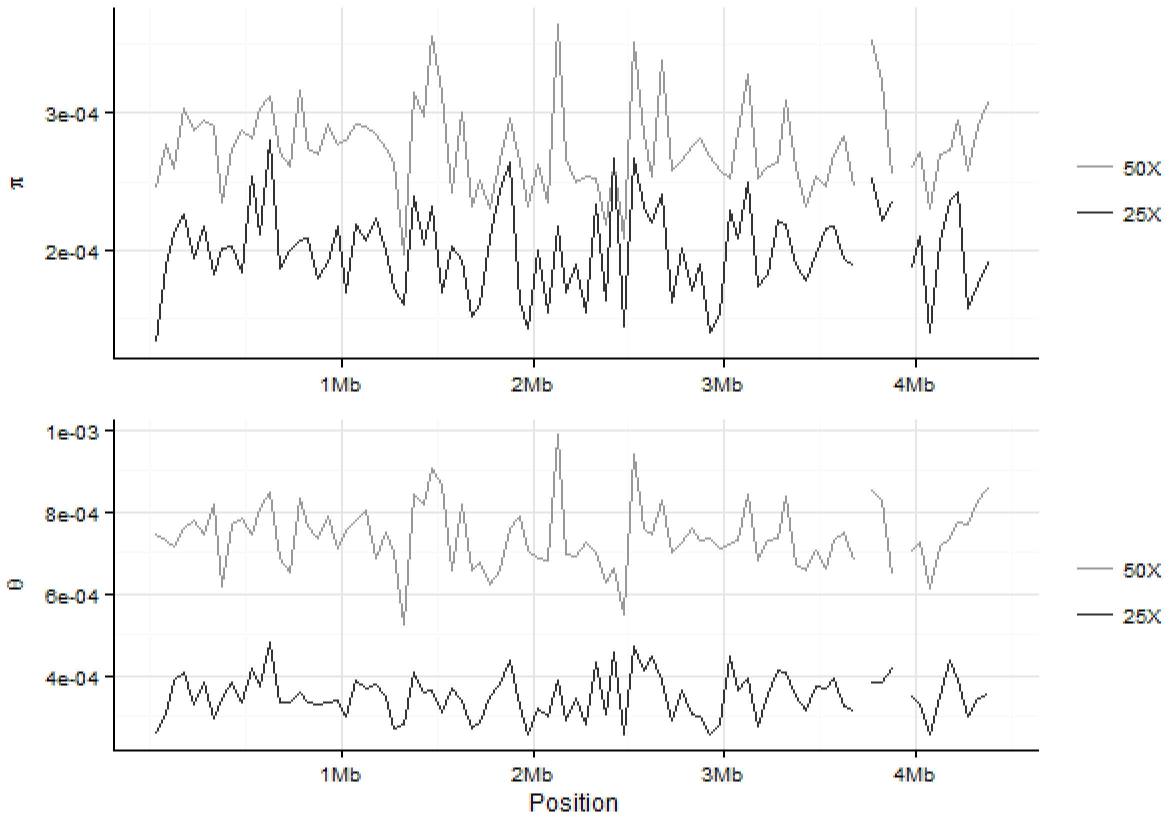

Fig 2. Effect of coverage on PoPoolation calculations. Calculations were performed using a window-size of 50,000, a minimum minor allele count of 2, and a pool-size of 10,000. Sequence data upon which calculations were performed were sub-sampled to a uniform coverage of 50X or 25X.

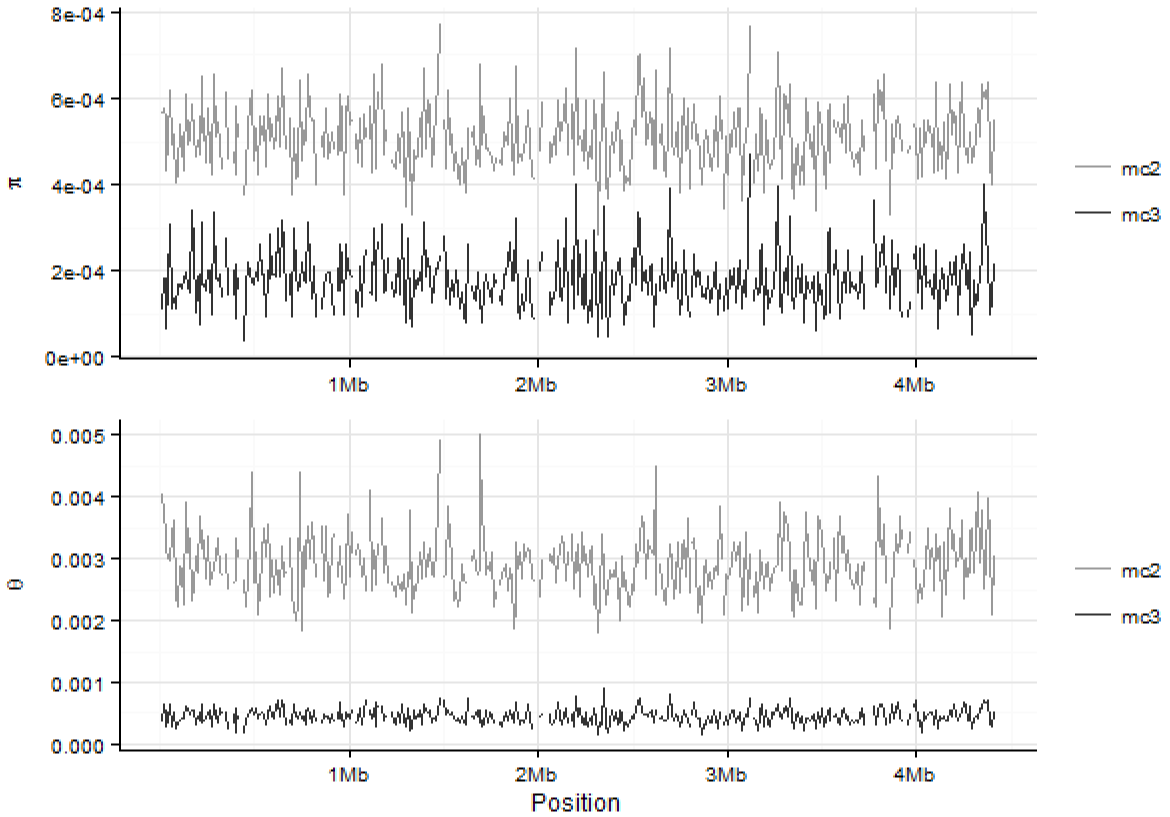

Fig 3. Effect of the minimum minor allele count on PoPoolation calculations. Calculations were performed using a window-size of 10,000 and a pool-size of 10,000. Calculations were performed with a minimum minor allele count of two or three as denoted by "mc" in the figure key.

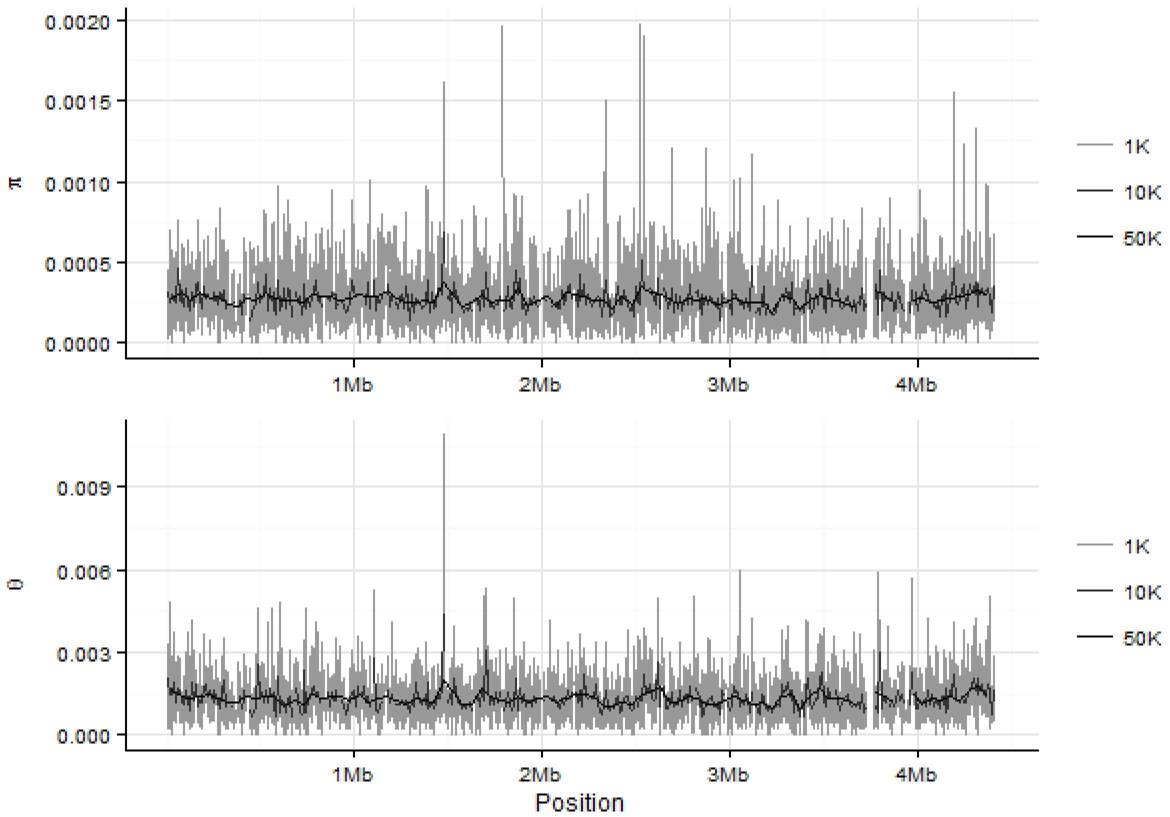

Fig 4. Effect of "window-size" on PoPoolation calculations. Calculations were performed using a pool-size of 10,000 and a minimum minor allele count of 2. Calculations were performed varying the window-size as indicated in the figure legend.
