# Supplementary figures and images for: Diversity of Mycobacterium tuberculosis across Evolutionary Scales
Source: PLoS Pathog. 2015 Nov 12;11(11):e1005257. doi: 10.1371/journal.ppat.1005257 (PMC4642946; doi:10.1371/journal.ppat.1005257)

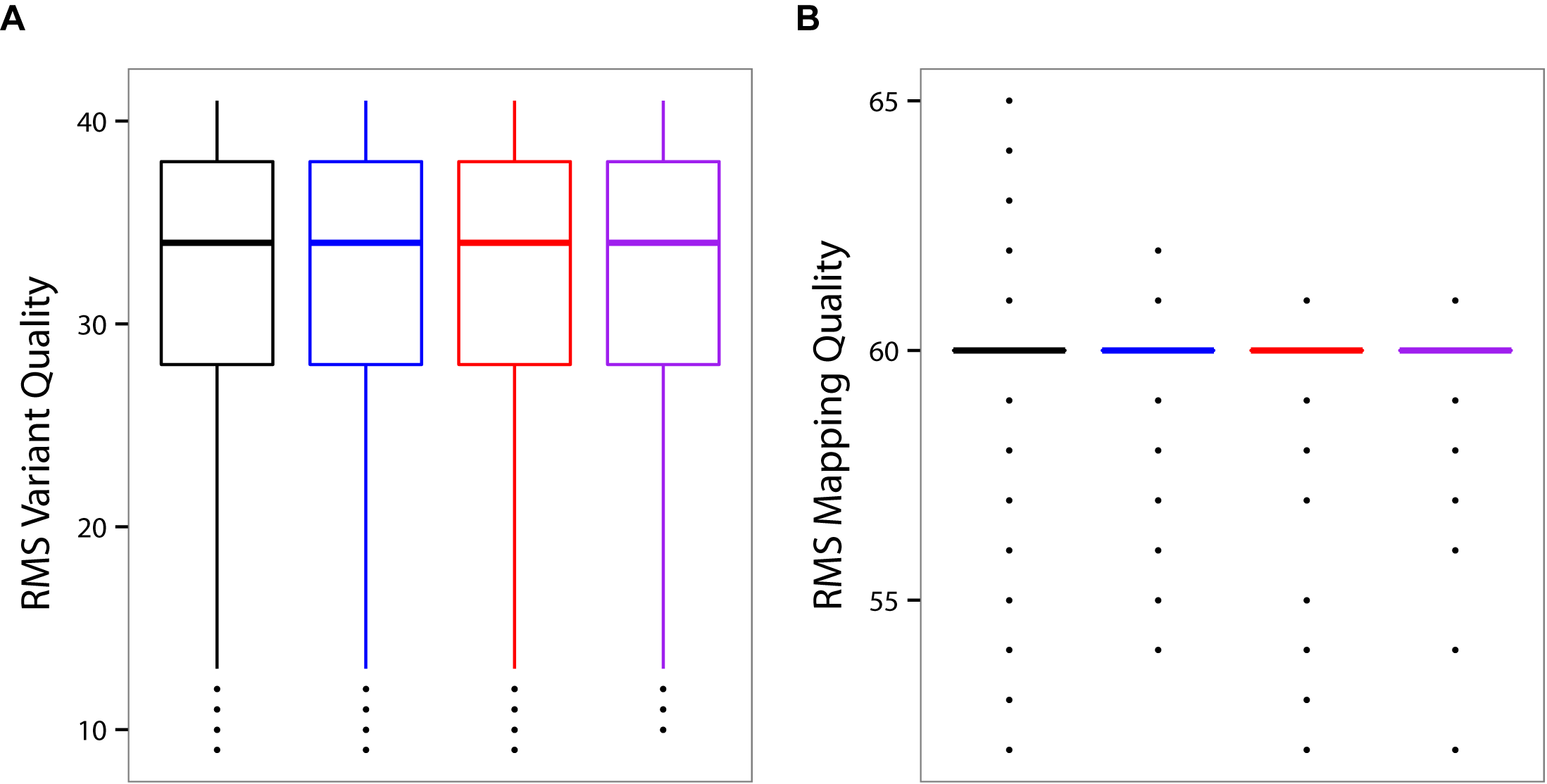

Supplement: S1 Fig — Box-and-whisker plots of (A) the root-mean-square (RMS) value of base qualities for variant alleles and (B) the RMS mapping quality for reads aligned at polymorphic sites in the reference-guided-assemblies (pooled across all within-host samples). RMS values were calculated with the python package pysamstats https://github.com/alimanfoo/pysamstats. Distributions are shown for polymorphisms occurring in any gene (black), TubercuList “conserved hypotheticals” (blue), TubercuList “lipid metabolism” (red), and COG:Q “secondary metabolites biosynthesis, transport, and catabolism” (purple). Upper and lower whiskers delineate highest values within 1.5 times the distance between the first and third quartiles. Outliers and plotted as points. (TIF) [file ppat.1005257.s013.tif]

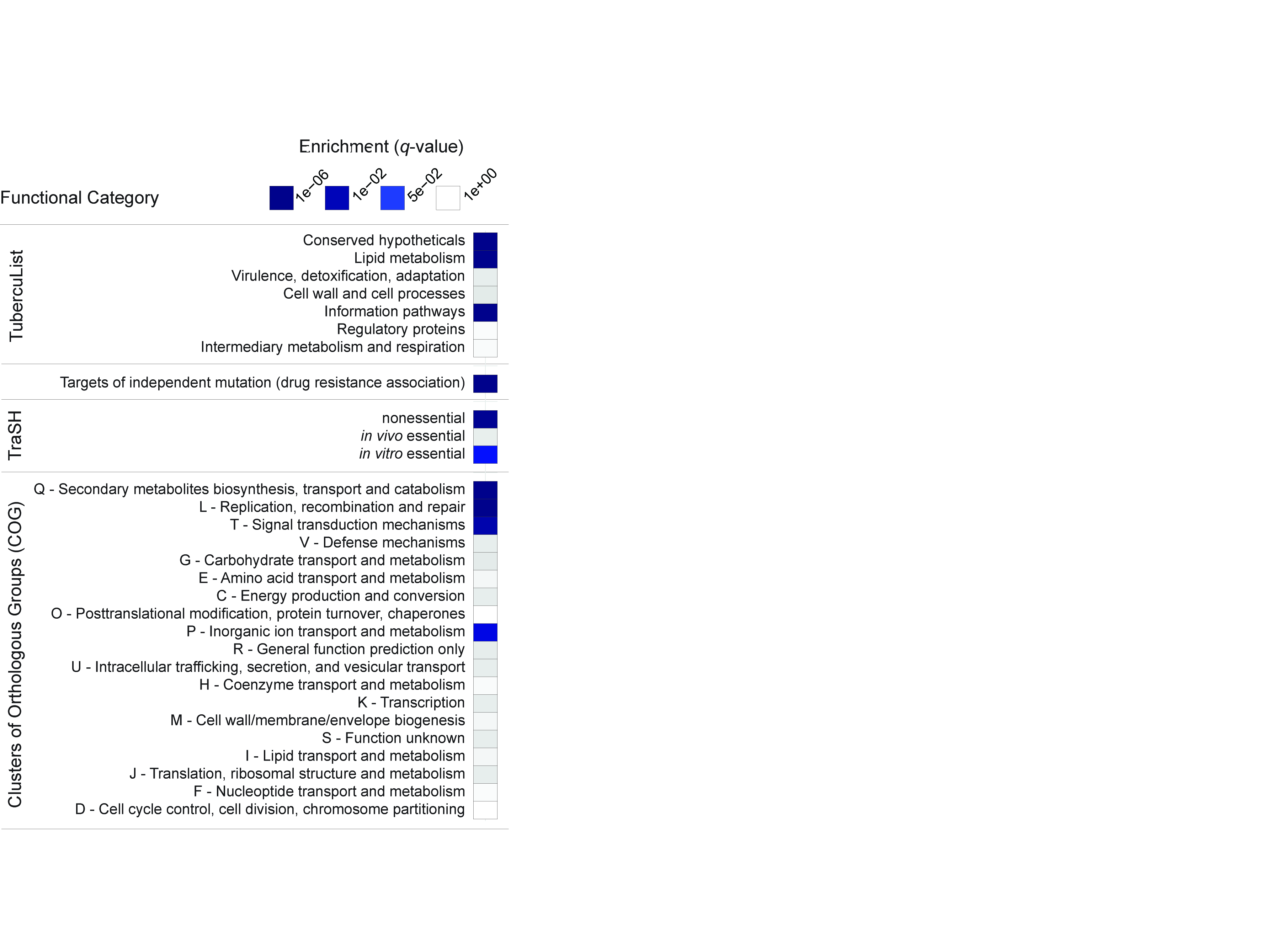

Supplement: S2 Fig — The total gene length covered by at least 75% of between-host strains were compared, and the top 5% of genes in the distribution were tested for overrepresentation of functional categories using a two-sided Fisher’s exact test. Genes which were not covered by at least 75% of strains for more than half of the total gene length were excluded. (TIF) [file ppat.1005257.s014.tif]

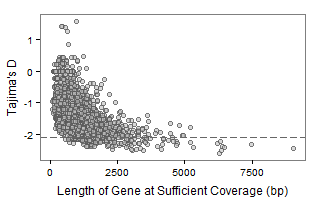

Supplement: S3 Fig — Each point corresponds to a gene in the H37Rv genome. Between-host, gene-wise values of TD are plotted against the length of the gene that was at sufficient coverage for the calculation. Genes for which less than half of the gene was covered by 75% of the strains have been excluded. The black dotted line marks the 5th percentile of TD. (TIF) [file ppat.1005257.s015.tif]
